# Potential of the World Network of Biosphere Reserves to advance the Kunming-Montreal Global Biodiversity Framework

**DOI:** 10.1093/nsr/nwaf449

**Published:** 2025-10-21

**Authors:** Hui Wu, Le Yu, Xiaoli Shen, Li Zhu, Ting Hua, Jianqiao Zhao, Yue Cao, Zhenrong Du, Tao Liu, Wenchao Qi, Shijun Zheng, Qiang Zhao, Jingsin Tan, Lijia How, Yixuan Li, Zufei Shu, Antonio Abreu, Keping Ma

**Affiliations:** Department of Earth System Science, Ministry of Education Key Laboratory for Earth System Modeling, Institute for Global Change Studies, Tsinghua University, Beijing 100084, China; Northeast Asia Biodiversity Research Center, College of Forestry, Northeast Forestry University, Harbin 150040, China; Department of Earth System Science, Ministry of Education Key Laboratory for Earth System Modeling, Institute for Global Change Studies, Tsinghua University, Beijing 100084, China; Ministry of Education Ecological Field Station for East Asian Migratory Birds, Beijing 100084, China; Institute of Carbon Neutrality, Tsinghua University, Beijing 100084, China; State Key Laboratory of Vegetation and Environmental Change, Institute of Botany, Chinese Academy of Sciences, Beijing 100093, China; State Key Laboratory of Vegetation and Environmental Change, Institute of Botany, Chinese Academy of Sciences, Beijing 100093, China; Industrial Ecology Programme and Department of Energy and Process Engineering, Norwegian University of Science and Technology, 7491 Trondheim, Norway; College of Land Science and Technology, China Agricultural University, Beijing 100083, China; Department of Landscape Architecture, School of Architecture, Tsinghua University, Beijing 100084, China; School of Information and Communication Engineering, Dalian University of Technology, Dalian 116024, China; Department of Earth System Science, Ministry of Education Key Laboratory for Earth System Modeling, Institute for Global Change Studies, Tsinghua University, Beijing 100084, China; Department of Earth System Science, Ministry of Education Key Laboratory for Earth System Modeling, Institute for Global Change Studies, Tsinghua University, Beijing 100084, China; Department of Earth System Science, Ministry of Education Key Laboratory for Earth System Modeling, Institute for Global Change Studies, Tsinghua University, Beijing 100084, China; Department of Earth System Science, Ministry of Education Key Laboratory for Earth System Modeling, Institute for Global Change Studies, Tsinghua University, Beijing 100084, China; School of Life Sciences, Tsinghua University, Beijing 100084, China; Xiuzhong College, Tsinghua University, Beijing 100084, China; Centre for Environmental Policy, Imperial College London, London SW7 2AZ, UK; Chebaling UNESCO MAB Reserve, Shaoguan 512500, China; Division of Ecological and Earth Sciences, Natural Sciences Sector, United Nations Educational, Scientific and Cultural Organization (UNESCO), Paris 75007, France; Secretary of the Man and the Biosphere (MAB) Programme, United Nations Educational, Scientific and Cultural Organization (UNESCO), Paris 75352, France; Northeast Asia Biodiversity Research Center, College of Forestry, Northeast Forestry University, Harbin 150040, China; State Key Laboratory of Vegetation and Environmental Change, Institute of Botany, Chinese Academy of Sciences, Beijing 100093, China

**Keywords:** biosphere reserves, protected areas, biodiversity, conservation, Kunming-Montreal Global Biodiversity Framework

## Abstract

As one of UNESCO’s three key site-based designations, the World Network of Biosphere Reserves (BRs) integrates conservation and development, setting it apart from traditional protected areas (PAs). Yet its conservation effectiveness and role in advancing the global biodiversity agenda remain underexplored. This evidence-based global assessment of BRs’ effectiveness and potential in supporting the Kunming-Montreal Global Biodiversity Framework (KMGBF) indicates that generally BRs maintained habitat quality not lower than that of PAs, with region-specific instances where BRs surpassed sites in IUCN Categories IV-VI. Including BRs—typically omitted from global conservation statistics—into conservation efforts increased terrestrial coverage for KMGBF Target 3 from 16.57% to 19.65%. With effective implementation, integration of BRs into the global area-based conservation network would produce measurable coverage gains across six KMGBF-linked opportunity templates, including +8.47% for Biodiversity Hotspots (per Target 1), +4.05% for Risk Ecoregions (per Target 2), +7.01% for Phylogenetic Diversity Hotspots (per Target 4), +7.25% for areas of high Traded Functional Diversity (per Target 5), +4.37% for regions of High Biomass Carbon (per Target 8), and +1.95% for globally Indigenous Lands (per Target 22). Based on integrated assessments of conservation value and coverage rate, 17 Udvardy’s Biogeographical Provinces were identified as post-2025 WNBR expansion priorities that align with the KMGBF and the Hangzhou Strategic Action Plan (2026–2035).

## INTRODUCTION

**Figure 1.**
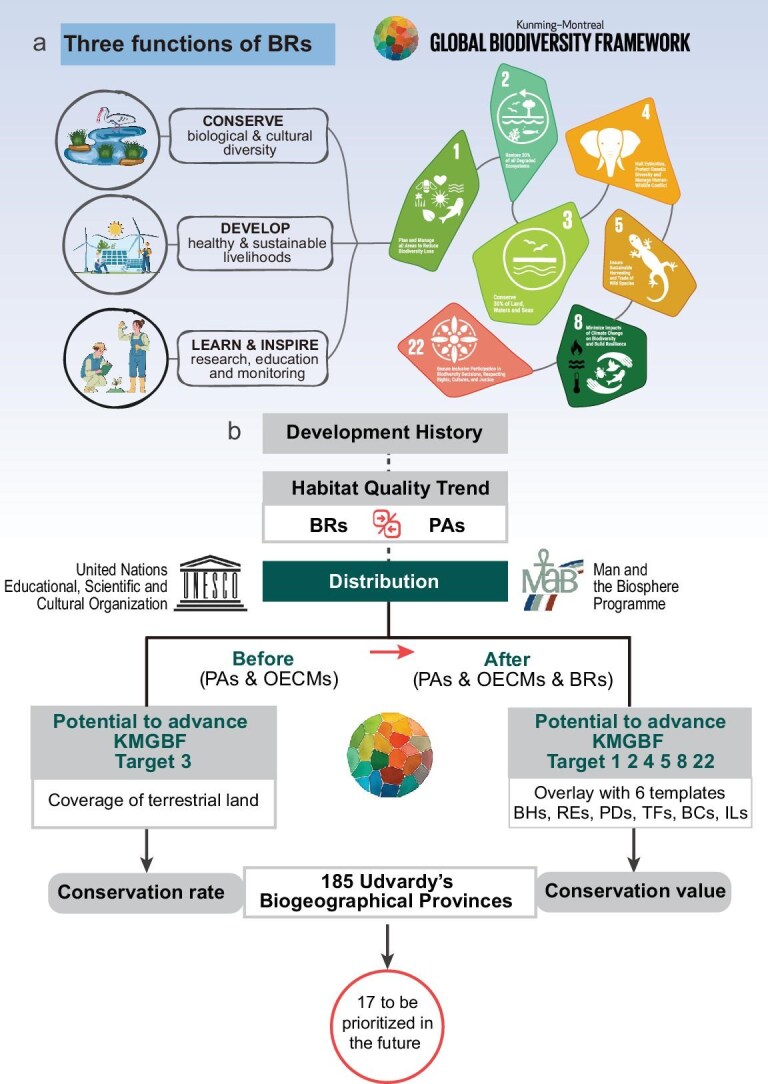
Flowchart of the study. Abbreviations: BRs, biosphere reserves; PAs, protected areas; OECMs, other effective area-based conservation measures; KMGBF, Kunming-Montreal Global Biodiversity Framework; BHs, REs, PDs, TFs, BCs, and ILs indicate the six target templates analyzed (details in Table S4).

Area-based conservation is central to the Kunming-Montreal Global Biodiversity Framework (KMGBF), which calls for spatially representative and effectively managed networks to safeguard biodiversity, ecosystem services, and human well-being. Within this global context, biosphere reserves offer a distinctive model of integrated conservation and development. In September 2025, the Fifth World Congress of Biosphere Reserves (the 5th WCBR) convened in Hangzhou, China. The congress adopted the Hangzhou Declaration and endorsed the Hangzhou Strategic Action Plan (2026–2035). This remarkable event traces back to 1968, when UNESCO’s Biosphere Conference laid the conceptual groundwork. In 1971 UNESCO launched the Man and the Biosphere (MAB) Programme [1] and in 1976 the first batch of biosphere reserves (BRs) were designated, thereby establishing the World Network of Biosphere Reserves (WNBR) [2–4]. As of 2024, 759 BRs across 136 countries together cover 7 667 281 km^2^—nearly the size of Australia. As a flagship program for sustainable development, the WNBR differs from traditional protected areas (PAs) by explicitly integrating biodiversity conservation with local development [5,6]. While PAs

typically prioritize ecological preservation through strict and exclusionary protection measures [7–10], BRs promote a more inclusive approach that embraces research, education, and sustainable use, fostering active community participation and equitable benefit-sharing (Fig. 1) [11,12]. In addition, unlike traditional PAs, which are largely state-led and governed with varying degrees of coordination, BRs benefit from being embedded in UNESCO’s global network, facilitating cross-border cooperation [13]. It is therefore worth considering the WNBR as a potentially valuable complement to PAs in implementing the global biodiversity agenda, particularly in light of the KMGBF’s emphasis on sustainable use and benefit-sharing [14].

Despite their potential, BRs remain marginal in global conservation policy and monitoring. Within the KMGBF, Target 3—which commits to protecting 30% of the planet’s land and marine areas by 2030 through an ‘ecologically representative, well-connected, and equitably governed system of protected areas and other effective area-based conservation measures (OECMs)’—does not explicitly include BRs, and they are therefore often overlooked in mainstream discussions. As of 2025, terrestrial and inland water protected areas account for a significant portion of the world’s land surface [15,16], yet BRs—despite their potential as effective conservation tools—are frequently excluded from the broader 30% conservation goal [17–19]. This exclusion arises from the limited recognition of BRs in global conservation efforts [20,21]. A significant body of literature critically examines their effectiveness, with some scholars contending that BRs function primarily as bureaucratic designations [22–24]. These critiques are grounded in regional case studies from countries such as Ireland [25], the Czech Republic [26], Central Europe [27], and various regions of Africa [28]. Therefore, demonstrating the effectiveness of BRs in conservation, especially in comparison to PAs, is essential for their potential to contribute to KMGBF Target 3 and other related targets.

Moreover, it is important to recognize that the WNBR may already support several dimensions of the KMGBF—not just the widely publicized ‘30 × 30’ commitment [29]. For instance, BRs are designed to protect and sustainably manage biodiversity across diverse ecosystems (per Target 1) [30], promote ecosystem restoration through adaptive management, reforestation, wetland rehabilitation, and other techniques (per Target 2) [31], provide refuges for endangered species and genetic diversity (per Target 4), and foster sustainable use of wild species through practices like eco-tourism, sustainable harvesting, and community-based management (per Target 5) [21,32–34]. Additionally, BRs offer nature-based solutions to mitigate climate change impacts, such as carbon sequestration, flood regulation, and habitat for species adaptation (per Target 8). They also actively involve local communities in decision-making, ensuring conservation efforts respect indigenous and local people’s rights, cultures, and needs (per Target 22) [35]. Yet, the extent to which the WNBR contributes to these targets remains unclear due to a lack of global-scale evidence [36].

As the 5th WCBR adopted the Hangzhou Declaration and the Hangzhou Strategic Action Plan, aligning the future trajectory of the WNBR with the KMGBF is both timely and necessary [37]. Therefore, we conducted this study (Fig. 1) to address three gaps in global BRs research: (Ⅰ) the absence of a global, quantitative evaluation of BRs effectiveness compared with PAs; (Ⅱ) the underexplored potential of BRs to advance KMGBF targets; and (Ⅲ) the lack of spatial guidance for future WNBR expansion based on global conservation priorities.

We first examined the development of the WNBR and its habitat trends from 1992 to 2020. By comparing these trends with the global PAs network and the trends of seven IUCN PAs categories, we assessed the effectiveness of the WNBR, which formed the basis for subsequent analysis. Next, we evaluated the potential of the WNBR to advance the 30 × 30 commitment (Target 3) and six additional KMGBF targets (Targets 1, 2, 4, 5, 8, and 22) by quantifying changes in the proportion of global terrestrial area and key priority areas that would be covered under a recognition scenario in which BRs are counted alongside PAs and OECMs. We operationalized this using six harmonized 1-km opportunity templates—Biodiversity Hotspots (Target 1), Risk Ecoregions (Target 2), Phylogenetic Diversity Hotspots (Target 4), Traded Functional Diversity Hotspots (Target 5), High Biomass Carbon (Target 8), and Indigenous Lands (Target 22)—and reported both realized coverage (PAs + OECMs) and potential uplift (PAs + OECMs + BRs). Importantly, we treat any BR–template overlap as an opportunity for future alignment, not as present, verified contribution. Finally, we outlined post-2025 expansion priorities for the WNBR by assessing the coverage rate of Udvardy’s Biogeographical Provinces and conservation value derived from layers aligned with KMGBF targets [38]. These priorities reflect the international scope and integrative mission of the BRs network, distinguishing them from the nationally defined, protection-oriented strategies typical of PAs. At this pivotal moment, with CBD COP16 concluding and the 5th WCBR approaching, this study provides new momentum and inspiration for the international cooperation between networks of both the WNBR and KMGBF.

## RESULTS

### Development of the WNBR

**Figure 2.**
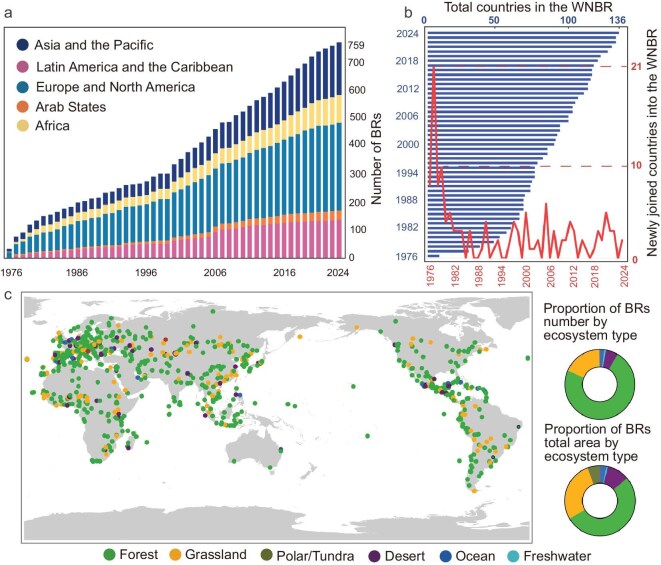
Development and distribution of the World Network of Biosphere Reserves. (a) Annual distribution of biosphere reserves across global regions (1976–2024). (b) Number of total and newly joined countries in the WNBR per year (1976–2024). (c) Global distribution of BRs and their proportion by number and total area across different ecosystem types as of 2024. Review drawing number: GS 京 (2025) 2215 号.

Since its establishment in 1976, the WNBR has expanded substantially, reaching 759 sites across 136 countries by 2024 (Fig. 2). Europe and North America host the largest share of BRs (41.31%), followed by Asia and the Pacific (24.60%) and Latin America and the Caribbean (18.18%), while Africa (13.10%) and the Arab States (4.28%) remain less represented (Fig. S1b). In terms of area, Latin America and the Caribbean account for nearly half of the total WNBR extent (46.70%), whereas the Arab States represent only 2.01% (Fig. S1c). The number of participating countries has steadily grown from 8 in 1976 to 136 in 2024, with Spain, Russia, and Mexico emerging as leading contributors (Fig. S2). Forest ecosystems dominate the network, representing nearly three-quarters of sites, while grasslands, deserts, tundra, and aquatic ecosystems remain less prevalent (Fig. 2c).

### Habitat quality trends in the biosphere reserves

**Figure 3.**
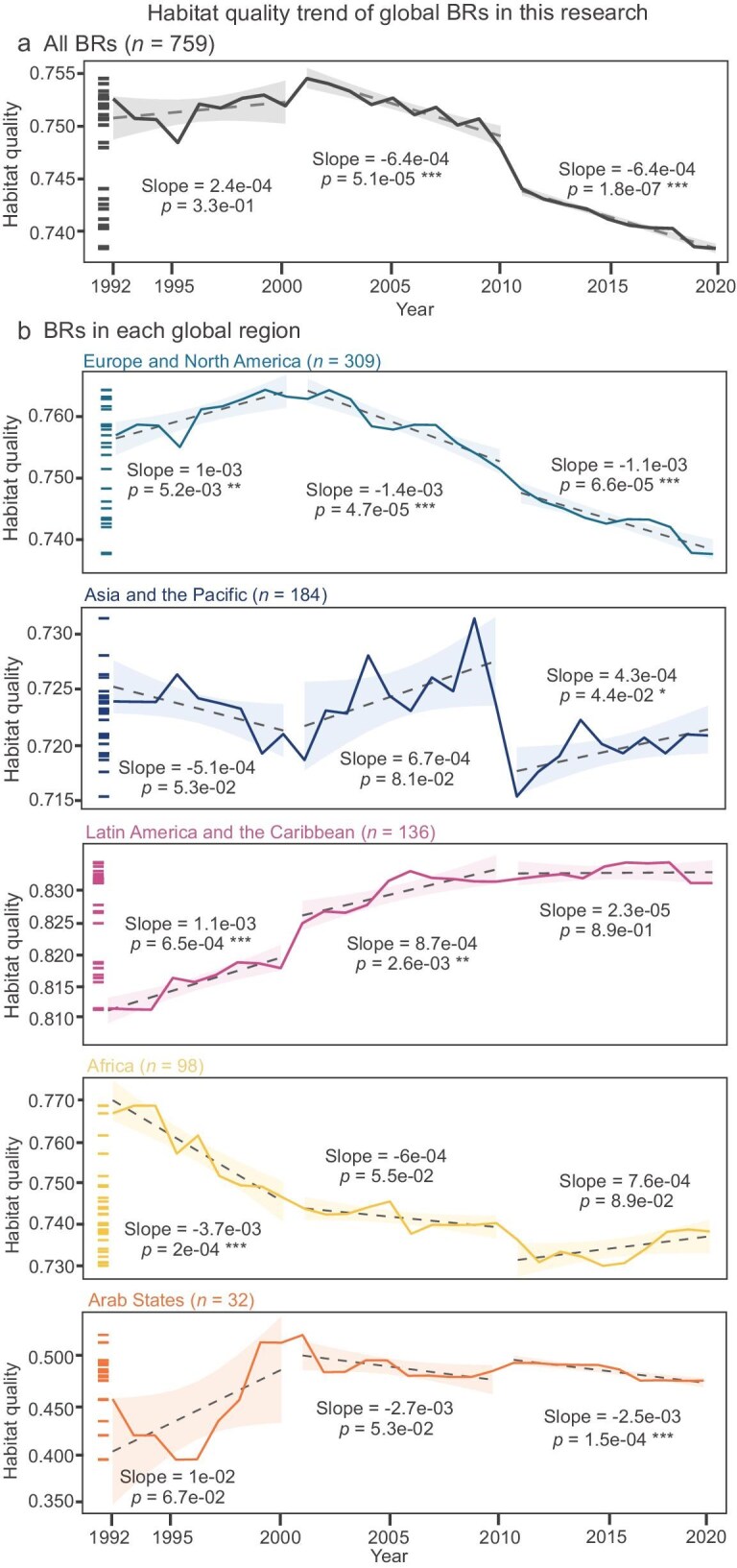
Trends in habitat quality from 1992 to 2020 for (a) all biosphere reserves (BRs, *n* = 759) and (b) BRs in different global regions (Europe and North America, Asia and the Pacific, Latin America and the Caribbean, Africa, Arab States). Slope p-values for each period (1992–2000, 2001–2010, and 2011–2020) are indicated in each panel to illustrate temporal trends. The significance levels are denoted as *p < 0.05; **p < 0.01; ***p < 0.001. Shaded areas represent 95% confidenceintervals, and dashed lines indicate the linear regression slopes. Small tick marks on the left side of each panel denote the distribution of annual mean habitat quality values over time.

Figure 3 illustrates habitat quality trends across BRs and PAs from 1992 to 2020, based on a global habitat quality map [39] derived using the InVEST model and 250 m resolution remote sensing data. Habitat quality is estimated by considering land use, anthropogenic threats, and habitat sensitivity. Annual mean habitat quality values were calculated for each BR, starting 5 years prior to their establishment, while PAs data were sourced from Zhao *et al.* [39] for comparability. The results show a decline in habitat quality across both BRs and PAs, although at varying rates (Fig. 3). Habitat quality in BRs remained relatively stable, decreasing from 0.753 in 1992 to 0.736 in 2020 (scale: 0 = lowest, 1 = highest). The rate of decline accelerated between 2000 and 2020 (slope = −6.4 × 10⁻⁴, *P* < 0.001) (Fig. 3a). Similarly, global PAs exhibited a dynamic trend, with habitat quality initially increasing (1992–2000: slope = 1.06 × 10⁻⁴, *P* < 0.01), then declining sharply (2000–2010: slope = −2.82 × 10⁻⁴, *P* < 0.001), and eventually showing a marked slowdown in decline (2010–2020: slope = −5.32 × 10⁻⁵, *P* = 0.082) (Fig. 3b). It is worth noting that this pattern may be influenced by differences in site size, number, and regional distribution.

Habitat quality trends among PAs varied by management category [39]. Strictly protected PAs (IUCN Categories I and II) maintained the highest habitat quality, though decline accelerated after 2000. Category IV and V PAs experienced substantial declines, while Category VI PAs, which allow for sustainable resource use, exhibited one of the sharpest declines. Habitat quality trends among BRs varied by region (Fig. 3b). Except for the Arab States, BRs in several other regions had overall habitat levels higher than Category V of PAs. In Latin America and the Caribbean, BRs maintained average habitat quality over the past 20 years at levels comparable to strictly protected areas (IUCN Categories I and III), and they declined less sharply than several other categories (e.g. IV–VI) during specific periods. Ecosystem-level results also showed contrasting patterns, with forests and grasslands exhibiting significant declines after the early 2000s, while deserts and polar ecosystems remained more stable until 2010 (Fig. S3). Grasslands showed a significant decline after the early 2000s (slope = –2.2 × 10⁻³, *P* < 0.001), while forests exhibited an initial slight increase followed by a marked decrease after 2010 (slope = –4.3 × 10⁻⁴, *P* < 0.01). Desert ecosystems experienced a strong decline during the 1990s (slope = –4.6 × 10⁻³, *P* < 0.001), with subsequent stabilization. These results suggest that BRs can, in some cases, maintain habitat quality at levels comparable to certain PA categories.

### The potential of the WNBR in advance 30 × 30 commitment

**Figure 4.**
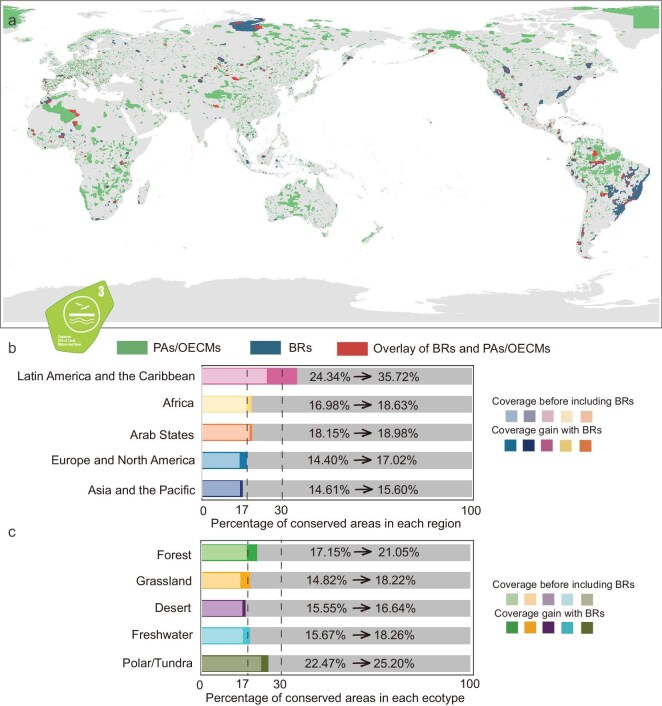
Coverage gains of conserved land with biosphere reserves (BRs) considered. (a) Global distribution of protected areas (PAs) and other effective area-based conservation measures (OECMs) in green, and BRs in blue. Areas where PAs/OECMs and BRs overlap are highlighted in red. (b) Percentage of conserved areas across different regions before and after including BRs (c) Percentage of conserved areas across different ecosystem types before and after including BRs. Transparent colors represent the conservation coverage before including BRs, while solid colors indicate the additional coverage gain achieved by incorporating BRs. Dashed lines at 17% and 30% mark the conservation area set by the Aichi Target and the KMGBF Target, respectively. Review drawing number: GS 京 (2025) 2215 号.

To assess the contribution of the WNBR toward achieving the 30 × 30 target (KMGBF Target 3), we spatially overlaid BRs’ boundaries with the existing global network of PAs and OECMs. We then quantified the additional conservation coverage contributed by BRs across regions and ecosystem types. When BRs were integrated with PAs and OECMs, conservation coverage for terrestrial areas increased from 16.57% to 19.65% (Fig. 4). Notably, overlap of BRs with existing PAs is primarily located in northern and central South America, northwestern Africa, and northern to central Asia. The inclusion of BRs in conservation networks resulted in significant improvements in the percentage of conserved areas across ecosystem types and regions, with some exceeding the 17% Aichi Target or the 30% KMGBF Target. Regionally (Fig. 4b), the most significant improvements occurred in Latin America and the Caribbean, where conservation coverage grew from 24.34% to 35.72%, surpassing the 30% KMGBF target. Coverage rose from 16.98% to 18.63% in Africa, and from 14.40% to 17.02% in Europe and North America, exceeding the 17% Aichi Target. Asia and the Pacific, along with the Arab States, experienced more modest improvements, with increases of 0.99% and 0.83%, respectively. Among ecosystem types (Fig. 4c), grasslands and freshwater achieved the 17% Aichi Target, with grasslands coverage rising from 14.82% to 18.22%, and freshwater coverage rising from 15.67% to 18.26%. Forests showed a notable increase, with an absolute gain of 3.90%. In contrast, deserts and polar/tundra ecosystems experienced more modest gains, with increases of 1.09% and 2.73%, respectively. The incorporation of BRs has led to an increase in conservation coverage across several countries, particularly in parts of Africa, South America, and Europe. Among the top 10 countries with the largest total area of PAs and OECMs, Brazil’s coverage increased from 29.94% to 49.70%, and Venezuela reached over 50% in both metrics (Fig. S4). Tanzania exhibited high national coverage after BRs were included, exceeding 40%. By contrast, countries such as Russia, Saudi Arabia, and the United States of America remained below 20% even after BRs were considered.

### Potential of the WNBR in advance of other KMGBF targets

To evaluate how the WNBR contributes to the KMGBF beyond area-based targets, we selected six global spatial templates representing key KMGBF action targets: (i) Biodiversity Hotspots (BHs) for species richness, (ii) Risk Ecoregions (REs) for ecosystem vulnerability, (iii) Phylogenetic Diversity Hotspots (PDs) for genetic diversity, (iv) Traded Functional Diversity Hotspots (TFs) for wild species trade, (v) High Biomass Carbon areas (BCs) for climate mitigation, and (vi) Indigenous Lands (ILs) for inclusive conservation. The six global conservation planning templates employed to assess the potential contributions of the WNBR to six KMGBF targets showed different spatial patterns (Fig. S5). BHs were primarily distributed between 40°N and 40°S, with prominent clusters in the Amazon Basin, Central Africa, Madagascar, Southeast Asia, and islands of the Pacific. REs spanned a wider latitudinal range, with a significant presence in both tropical and temperate zones, notably across North America, Central Europe, Central Africa, and Southeast Asia. PDs and TFs were mainly found within equatorial regions with core areas in western Amazonia, the Congo Basin, and Sundaland. BCs were distributed extensively across the Amazon Basin, Central Africa, and Southeast Asia. ILs were widely dispersed across all continents, with notable concentrations in North and South America, Australia, and parts of Asia and Africa.

Spatial overlay analysis (Fig. S6a) revealed that most land areas were covered by one (41.99%) or two (18.05%) template layers. Areas covered by all six conservation templates account for only 0.68% of global terrestrial land, primarily concentrated in Southeast Asia, with some distribution in tropical regions of Africa and the Americas. Among the six global conservation planning templates (Fig. S6b), REs covered the largest area followed by ILs. However, 46.15% of REs and 51.37% of ILs did not overlap with any other templates. In contrast, PDs showed the highest overlap: 3.90% of PDs intersected with all the other five templates, and 10.38% with four. BHs also exhibited high overlap, with 3.67% overlapping with all the other five templates and 10.18% with four.

**Figure 5.**
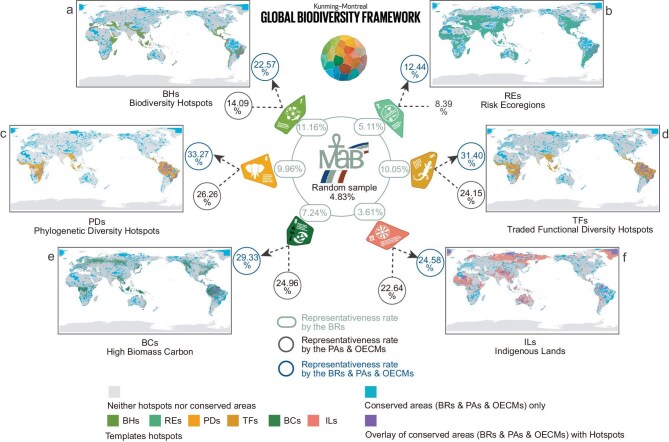
Global representativeness rates of biosphere reserves (BRs), protected areas (PAs), and other effective area-based conservation measures (OECMs) for six target templates under the Kunming-Montreal Global Biodiversity Framework (KMGBF). (a) Biodiversity Hotspots (BHs). (b) Risk Ecoregions (REs). (c) Phylogenetic Diversity Hotspots (PDs). (d) Traded Functional Diversity Hotspots (TFs). (e) High Biomass Carbon (BC). (f) Indigenous Lands (ILs). Percentages within different colored circles reflect the proportion of each template covered by the respective conservation networks (green for BRs, grey for PAs & OECMs, blue for BRs & PAs & OECMs). The percentages shown for BRs (green) and for PAs & OECMs (gray) do not add up to the combined percentages (blue), because of spatial overlaps among these conservation networks. Purple shading highlights overlaps between conserved areas (BRs, PAs & OECMs) and the templates, while blue shading represents conserved areas only. Coverage rate of BRs as random sample (4.83%) provides a baseline for comparison. Review drawing number: GS 京 (2025) 2215 号.

We evaluated spatial representativeness using three metrics: the proportion of each template area within BRs’ boundaries, compared to the global terrestrial area of BRs (4.83%), which serves as a baseline representing random spatial distribution; the cumulative representativeness of the mainstream global network of PAs and OECMs; and the cumulative representativeness of the combined BRs–PAs–OECMs network. Figure 5 illustrates these representativeness rates for six opportunity templates that are potentially aligned with KMGBF Targets 1, 2, 4, 5, 8, and 22, highlighting the potential of the WNBR to support global biodiversity conservation. The results showed that compared to a random sample (4.83%), the current global distribution of the WNBR protected 11.16% of BHs, 5.11% of REs, 9.96% of PDs, 10.05% of TFs, and 7.24% of BCs, demonstrating an advantage in supporting the corresponding five KMGBF targets. Only the protection rate of ILs (3.61%) was lower than the random sample (Fig. 5). Taking BRs into consideration could remarkably enhance the representativeness rates of the global conserved areas network for target templates. While PAs and OECMs alone contributed coverage to templates such as BHs (14.09%), PDs (26.26%), and TFs (24.15%), the combined network of BRs, PAs, and OECMs achieved much higher representativeness rates of 22.57%, 33.27%, and 31.40%, respectively.

In Latin America and the Caribbean, BRs covered 30.72% of BHs. Incorporating BRs into the conserved areas network increased BH protection in the region from 15.63% to 40.45%, and in the Arab States from 15.93% to 20.52% (Fig. S7). For REs, the inclusion of BRs raised protection levels to 26.89% in Latin America and the Caribbean, 12.19% in Africa, 12.58% in the Arab States, 9.14% in Europe and North America, and 8.88% in Asia and the Pacific (Fig. S8). In the Arab States and Europe and North America, where PDs were sparsely distributed, the inclusion of BRs yielded only marginal gains in coverage. In Europe and North America, PD coverage increased only slightly from 14.76% to 15.77%, while no improvement was observed in the Arab States due to a lack of spatial overlap between BRs and the PDs template. In contrast, in regions with higher PD concentrations, incorporating BRs raised PD coverage to 44.05% in Latin America and the Caribbean, 25.47% in Africa, and 15.23% in Asia and the Pacific (Fig. S9). TFs showed distributional characteristics comparable to those of PDs (Fig. S10). In Latin America and the Caribbean, where BCs had the highest regional coverage, incorporating BRs into the conserved areas network increased BCs’ protection from 41.08% to 49.11% (Fig. S11). After incorporating BRs, global conserved areas exhibited higher representativeness for ILs, particularly in Latin America and the Caribbean and the Arab States, where ILs were already well protected. In both regions, ILs’ representativeness exceeded 50% after including BRs (Fig. S12). However, in the Arab States, due to the spatial overlap between BRs and existing PAs or OECMs, the inclusion of BRs only increased ILs’ protection by 2.33%, despite BRs alone representing 10.03% of ILs.

### Post-2025 expansion priorities for the WNBR

**Figure 6.**
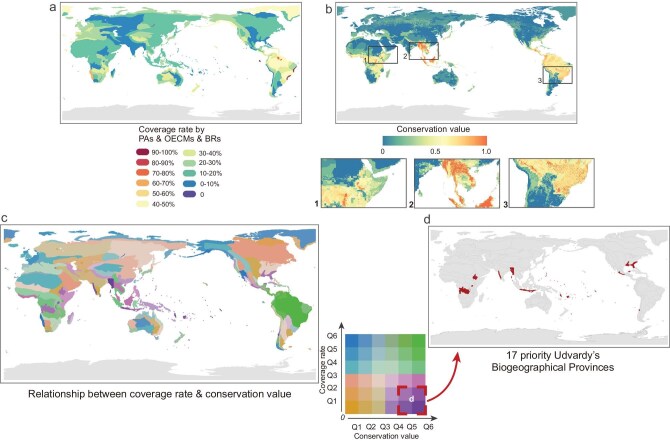
Post-2025 expansion priorities for the WNBR. (a) Udvardy’s Biogeographical Provinces’ coverage rate by biosphere reserves (BRs), protected areas (PAs), and other effective area-based conservation measures (OECMs). (b) Global map of Conservation Value Index. (c) Spatial relationship between coverage rate and conservation value (based on combined coverage of BRs, PAs, and OECMs). (d) Seventeen priority Udvardy’s Biogeographical Provinces (in red) identified for the post-2025 expansion of the WNBR. Q1 to Q6 represent coverage rate and conservation value index from lowest to highest, corresponding to the bottom 1/6 (Q1), 2/6 (Q2), 3/6 (Q3), 4/6 (Q4), 5/6 (Q5), and top 1/6 (Q6). Review drawing number: GS 京 (2025) 2215 号.

Post-2025 expansion priorities for the WNBR were identified based on two dimensions: coverage rate and conservation value. Although the aim was to guide the expansion of BRs, coverage rate was assessed using the combined spatial coverage of PAs, OECMs, and BRs, to reflect the overall conservation saturation of each Udvardy’s Biogeographical Province. Conservation value was quantified using a Conservation Value Index derived from six global conservation templates. Figure 6 presents the spatial distribution of conservation priorities, highlighting key provinces most in need of WNBR expansion.

Higher coverage levels of BRs, PAs, and OECMs across biogeographical provinces were observed in parts of South America, southern Africa, and Australia (Fig. 6a). Provinces with the lowest coverage (<10%) were widely distributed across Southeast Asia, Central Asia, and portions of North America. High conservation value regions (index >0.8) were primarily located in tropical areas (Fig. 6b), including northern and central South America, Central Africa, and Southeast Asia. These areas fell mostly within 20°N–20°S latitude. Spatial relationships between coverage rate and conservation value were summarized in a 6 × 6 quantile matrix, in which provinces were binned into sextiles for each variable (Q1–Q6), with Q1 and Q6 denoting the lowest and highest empirical quantiles (i.e. bottom and top one-sixth), respectively (Fig. 6c).

Seventeen Udvardy’s Biogeographical Provinces—i.e. high conservation value (Q5–Q6) but low existing coverage (Q1–Q2)—were therefore identified as priorities for post-2025 WNBR expansion (Fig. 6d, Table S1). Strategically targeting these ecologically significant but underprotected regions would directly address persistent conservation blind spots, accelerating progress toward KMGBF Goal A by reinforcing the global ecological network’s integrity, connectivity, and resilience. For instance, the Guerreran biogeographical province was identified due to its low level of coverage rate (5.74%) and intermediate conservation value ranking (0.47). Provinces like the Ethiopian Highlands and Lesser Sunda Islands had both low coverage rates (8.14% and 12.51%) and medium conservation value (>0.33), making them key targets for expansion. Analysis of the mean Human Footprint (HFP) scores across biogeographical provinces revealed that several of the identified provinces exhibited relatively high human footprint pressures (Fig. S13). For instance, the Micronesian reached an HFP score of 11.92, and the Austroriparian 9.29 (Table S1). In these provinces, the expansion of conserved areas might be more feasibly achieved through the designation of BRs, whose concepts and management frameworks may be more adaptable under conditions of higher human influence than conventional PAs and OECMs.

## DISCUSSION

### Recognizing BRs can help close KMGBF Target 3 gaps

The role of the WNBR in supporting the SDGs, particularly those related to biodiversity, climate action, and sustainable communities, has long been recognized [40–43]. However, this recognition has also led to skepticism regarding the effectiveness of BRs in achieving strong conservation outcomes, with some viewing them as a more flexible or lenient form of area-based protection. Contrary to these long-standing assumptions, our analysis reveals that BRs have demonstrated resilience in maintaining habitat quality that is, in many cases, comparable to that of conventional PAs [25,26]. Despite their integrative design and emphasis on human–nature coexistence, BRs have not compromised core conservation outcomes [27]. These findings are consistent with recent studies emphasizing the effectiveness of less restrictive protection models, particularly when supported by strong local engagement and adaptive governance [12,28]. This evidence challenges the binary notion that conservation success is solely tied to strict exclusion, reinforcing the idea that multifunctional landscapes, like those promoted under the MAB Programme, can deliver meaningful ecological outcomes [44].

Building on the understanding of BRs’ effectiveness in conservation, it is crucial to address the current shortfalls in achieving Target 3. As of July 2025, among the 137 Parties that have submitted National Biodiversity Strategies and Action Plans (NBSAPs) or national targets, more than half have neither incorporated explicit commitments to protecting 30% of their land and sea nor clarified their intentions regarding this goal [45]. Additionally, 61 countries have yet to submit any plan addressing the target. While the KMGBF sets this as a global objective, the countries that have omitted the 30% target from their plans collectively account for nearly one-third of the Earth’s land area. Notably, this group includes several mega-diverse nations with high concentrations of biodiversity, such as Mexico, Indonesia, Malaysia, Peru, the Philippines, South Africa, and Venezuela. These facts underscore growing concerns about the potential for another decade of international shortfalls in biodiversity conservation. The WNBR could play a remarkable role in achieving the 30 × 30 targets. Empirically, the integration of BRs alongside PAs and OECMs meaningfully increased global terrestrial conservation coverage, with some regions—such as Latin America and the Caribbean—surpassing the 30% KMGBF Target 3 [46–48].

### BRs enable multi-target opportunities under the KMGBF

In light of the role of BRs in addressing global conservation shortfalls, their alignment with the KMGBF’s integrated and inclusive approaches further emphasizes their potential to advance biodiversity goals. The WNBR, structurally aligned with the KMGBF’s focus on ecosystem-based management, can leverage its governance framework and participatory model to contribute to a range of targets. Empirical evidence shows that BRs have significantly improved conservation outcomes across various ecosystem types, reinforcing Target 1 and Target 2 by supporting biodiversity-inclusive spatial planning and restoration in diverse biogeographical contexts [49,50]. When BRs were considered in combination with existing PAs and OECMs, substantial gains were observed in the representation of BHs and REs, particularly in tropical regions such as Latin America and the Caribbean, and sub-Saharan Africa. The spatial overlay analysis indicates that BRs intersect substantially with PDs (per Target 4) and with areas of TFs (per Target 5)—notably in western Amazonia, the Congo Basin, and Sundaland [51]—while also offering potential contributions to Target 8 by bolstering ecosystem resilience and carbon storage in climate-vulnerable landscapes across the Global South. In addition, the WNBR’s participatory model intrinsically supports the equity aims of Target 22, reinforcing its value for inclusive biodiversity governance. Moreover, BRs also support Targets 9, 10, and 11 by promoting biodiversity-based livelihoods [52,53], sustainable land-use practices, and nature’s contributions to people—particularly through local knowledge systems and community engagement [54]. By enhancing spatial representativeness, bridging policy goals, and promoting co-benefits across ecological and socio-economic dimensions, the WNBR should be recognized not merely as a legacy SDG-aligned mechanism, but as a vital, underutilized asset in accelerating KMGBF implementation. Greater attention to this dual role can enhance global policy coherence and substantially contribute to the achievement of 2030 biodiversity and sustainability targets [55,56].

### Priority biogeographical provinces indicate where BRs add the most value

The record number of BR submissions in 2025—including entries from five first-time countries—signals a renewed political momentum and international endorsement of the BRs’ model. With over 30 proposals currently under review, many countries are aligning their domestic strategies with global sustainability commitments through the WNBR framework. However, despite this progress, the current distribution of BRs remains uneven. Europe and North America host the largest number of BRs, while tropical and subtropical regions—particularly in Central Africa, Southeast Asia, and some countries in northern South America—harbor areas of significantly higher conservation value but remain underrepresented. These regions overlap with biodiversity hotspots and offer crucial opportunities to advance KMGBF Target 3, especially through community-oriented, nature-positive designations [19,57–59]. The identification of 17 priority Udvard’s Biogeographical Provinces—defined by their high conservation value and limited existing coverage—highlights the need for a more ecologically representative and socially equitable WNBR. Although institutional readiness varies, some regions like Congo Woodland and the Lesser Sunda Islands are in countries already active in the MAB framework, while others may require enhanced international cooperation, governance innovation, or capacity-building support [60].

Furthermore, many priority provinces face elevated anthropogenic pressures, as evidenced by high Human Footprint scores. In these contexts, BRs offer a flexible and adaptive alternative to conventional PAs and OECMs, particularly due to their emphasis on participatory governance and socio-ecological integration. The suitability of BRs in human-dominated landscapes positions them as a key tool for addressing GBF targets that intersect conservation, sustainable development, and resilience-building, especially in regions where rigid protection frameworks may face social or political constraints. To enhance the role of the WNBR in the KMGBF implementation process, future network growth should not only focus on increasing numerical representation but also improving ecological representativeness, connectivity, and conservation performance [61]. This requires spatially informed prioritization, integration with NBSAPs, and strengthened collaboration with other area-based mechanisms. As BRs continue to bridge science, policy, and practice, their expanding role within global conservation efforts can serve as a model for integrating biodiversity conservation with human well-being in an era of rapid global change [62,63].

### Limitations and future work

This study demonstrates that the inclusion of BRs in conservation networks significantly enhances the spatial coverage and ecological representativeness of conserved areas, thereby supporting multiple targets within the KMGBF. However, several limitations should be acknowledged. First, the lack of detailed boundary shapefiles for many BRs posed a challenge in accurately delineating reserve areas. While vector data for 73.58% of the total area of BRs were available and boundaries for the remaining reserves were approximated using circular buffers, this methodology introduces some uncertainty in the total area estimates and the precise spatial extent of the reserves. Second, habitat quality trend comparisons between BRs and PAs are indicative rather than conclusive, as differences in sample size, spatial distribution, and area could influence the observed trends in habitat quality and confound assessments of relative conservation performance. Third, it is important to note that the template overlaps reported in this research do not represent realized KMGBF target delivery. Instead, they indicate potential opportunities for alignment, conditional upon the formal recognition of BRs and their effective implementation within national and global biodiversity strategies. Fourth, the prioritization analysis is based on general ecological criteria and not tailored to BR-specific features. Future work could consider BRs’ unique governance, zonation, and development mandates to better guide their strategic expansion. Despite these limitations, this study contributes significantly to the growing body of knowledge on BRs and their potential to meet global conservation targets. By highlighting the crucial role of BRs in expanding and enhancing global conserved area networks, the findings underscore the importance of integrating BRs into global conservation frameworks, providing a solid foundation for future WNBR development and conservation planning, paving the way for more effective and equitable biodiversity protection worldwide.

## METHODS

### Data collection of BRs, PAs, and OECMs 

Information on the WNBR was obtained from UNESCO’s MAB official website and biodiversity portal. The dataset includes latitude, longitude, year of designation, total area, country, and regional group (Africa, Arab States, Asia and the Pacific, Europe and North America, Latin America and the Caribbean) for all 859 BRs. Boundary shapefiles covering 73.58% of the total area of BRs were compiled from the World Database on Protected Areas (WDPA) [64] and Palliwoda *et al.* [65] and, supplemented by datasets, our team were able to gather and harmonize from multiple external repositories. Notably, the WDPA includes some sites labeled as MAB that are not officially listed by the MAB program, which were excluded from our study. For the remaining BRs, circular buffers were generated around their central coordinates, with buffer sizes determined based on terrestrial area data from the UNESCO MAB website. To classify BRs into ecosystem types, the dominant WWF Biome [66] (i.e. the biome with the largest area proportion within each BR) was identified. BRs were then categorized into six ecosystem groups based on the classification criteria outlined in Table S2: Forest, Grassland, Polar & Tundra, Desert, Ocean, and Freshwater. PA and OECM boundaries were obtained from the WDPA and WDOECM databases [64]. PAs labeled as MAB in the DESIG_ENG field and those marked as ‘proposed’ in the STATUS field were excluded. Given the incomplete representation of China’s PAs in the WDPA and WDOECM databases, additional boundary data for local PAs, including national parks and nature reserves, were incorporated. Following data processing, the terrestrial and inland water PAs and OECMs coverage in our study was 16.57%, slightly lower than the 17.54% reported by the WDPA and WDOECM databases as of 2025.

### Trend analyses for habitat quality

We overlaid the boundaries of BRs with our global habitat quality map to calculate the annual mean habitat quality values within each BR from 1992 to 2020. The 250 m resolution habitat quality map used in this study was generated using the Integrated Valuation of Ecosystem Services and Trade-offs (InVEST) model [67], which estimates habitat quality based on global annual remote sensing data, reflecting the capacity of habitats to support species and ecological functions. Land cover data were obtained from the ESA Climate Change Initiative Land Cover (ESA CCI LC) dataset, with a reported accuracy of 70.5% for 2020 [68,69]. Model parameters were calibrated using empirical estimates synthesized from 92 peer-reviewed publications, enhancing ecological validity and generalizability across biomes.

Habitat quality was evaluated starting 5 years before each BRs’ establishment, rather than from the designation year, to account for preparatory efforts such as restricting human activities and implementing management measures that commonly precede official designation. By this method, we obtained time series data for each BRs’ habitat quality, starting either from 1992 (for BRs established in 1997 or earlier) or from 5 years before official designation (for BRs established after 1997) through 2020. Based on the annual mean habitat quality of each BR, we calculated the global, regional, and ecosystem-specific trends in BRs’ habitat quality. The period 1992–2020 was divided into three sub-periods (1992–2000, 2001–2010, and 2011–2020). Separate linear regressions were fitted for each sub-period to capture potential phase-specific trends in habitat quality change and to allow comparison across time intervals. To further evaluate the conservation performance of BRs, we compared their habitat quality trends with those of PAs reported in Zhao *et al.* [39], which includes all global PAs as well as those categorized by the IUCN management categories (I–VI and unspecified, Table S3): I—Strict Nature Reserve or Wilderness Area; II—National Park; III—Natural Monument/Feature; IV—Habitat/Species Management Area; V—Protected Landscape; VI—Protected Area with Sustainable Use of Natural Resources; Unspecified.

### Definition and KMGBF alignment of the six global templates

To appraise how the WNBR could potentially align with the KMGBF, we curated six global spatial templates addressing species biodiversity, ecosystems, genetic diversity, wild species trade, climate change impacts, and indigenous peoples’ rights (Table S4), each mapped to one KMGBF action target (Targets 1, 2, 4, 5, 8, and 22). Each template was rasterized at 1-km resolution as a binary mask (inside/outside), thereby enabling coverage and representativeness calculations. For each template we report: (i) realized coverage (PAs + OECMs, already counted in official accounting); and (ii) potential coverage under a recognition scenario where qualifying BRs are counted alongside PAs/OECMs and appropriately managed. The difference represents uplift potential.

To appraise potential alignment with KMGBF Target 1, we treated Biodiversity Hotspots (BHs) [70] as a 1-km opportunity template. BHs are regions that are both biologically rich and highly threatened; although the 36 BHs occupy ∼2.5% of land, they hold over half of endemic plant species and ∼43% of endemic vertebrates. For Target 2, we used Risk Ecoregions (REs) [71] as an opportunity template, representing ecoregions with severe habitat conversion and low PA coverage that are therefore at elevated risk. For KMGBF Target 4, which aims to halt extinction, protect genetic diversity, and manage human–wildlife conflict, we used Phylogenetic Diversity Hotspots (PDs) [72] as an opportunity template, comprising global phylogenetic diversity maps for birds and mammals. These maps are based on comprehensive, time-calibrated species-level phylogenetic trees. The bird tree, derived from Jetz *et al.* [73] with a Hackett backbone, includes 9561 species, while the mammal tree from Upham *et al.* [74] includes 5231 species. The top 10% of land areas with the highest phylogenetic diversity were identified for mammals (PD threshold: 2594.615) and birds (PD threshold: 9551.888). Their overlap defined PDs, covering 12.86% of global terrestrial areas.

To address KMGBF Target 5, which focuses on sustainable harvesting and trade of wild species, the template Traded Functional Diversity Hotspots (TFs) for birds and mammals [72] was used as an opportunity template. Functional trait data were sourced from Wilman *et al.* [75] and assigned at the species level, encompassing four key traits: body mass, dietary composition, foraging strata, and activity period. These traits capture essential aspects of Eltonian niche space, reflecting resource use and ecosystem interactions. The top 10% of land areas with the highest traded functional diversity were identified for mammals (TF threshold: 0.0591) and birds (TF threshold: 0.1117). Their overlap defined TF hotspots, covering 14.47% of global terrestrial areas. For KMGBF Target 8, which aims to minimize climate change impacts on biodiversity and enhance resilience, the template High Biomass Carbon (BC) [76] was used as an opportunity template. BC identifies regions with high aboveground and belowground biomass carbon density across vegetation types. Conserving these areas preserves carbon storage, preventing emissions from land-use changes such as deforestation or agricultural expansion, thereby reducing atmospheric CO_2_ and mitigating global warming [77]. For Target 22, which aims to ensure inclusive participation in biodiversity decisions while respecting rights, cultures, and justice, the template Indigenous Lands (ILs) [78] was used as an opportunity template. Indigenous communities hold ancestral rights to lands they have managed sustainably for generations. Ensuring their participation protects their rights, respects their knowledge, and enhances ecosystem resilience through practices like rotational farming and controlled fishing.

To quantify the spatial convergence across multiple conservation priority layers, a spatial meta-analysis was conducted by overlaying six global conservation planning templates. Each cell was assigned a cumulative value ranging from 0 to 6 based on the number of overlapping template layers it belonged to. A stacked bar chart was used to visualize the internal composition of each template in terms of its frequency of overlap with the remaining five templates.

### Potential of the WNBR in advancing KMGBF targets

To assess the potential of the WNBR in advancing KMGBF targets, we calculated three representativeness rates. The first type evaluates the representativeness of global BRs for the six templates and compares it to the global terrestrial area percentage covered by BRs, which serves as a random sample. This assesses the effectiveness of BRs’ spatial distribution. The second type evaluates the representativeness of the global network of PAs and OECMs for the same templates. The third type calculates the representativeness of the combined global network of BRs, PAs, and OECMs for the templates. By comparing the second and third rates, we assessed the potential of the WNBR in advancing the KMGBF targets.

### Identification of region expansion priorities for the WNBR

We identified post-2025 expansion priorities for the WNBR from two dimensions. The first dimension is coverage rate, based on Udvardy’s Biogeographical Provinces, which consist of 193 units (185 included in this analysis) developed by the IUCN under the UNESCO MAB Programme to classify biotic areas for conservation. To assess conservation saturation across regions, we calculated the proportion of each Udvardy’s Biogeographical Province covered by conserved areas, including global PAs, OECMs, and BRs. Although the goal was to guide future expansion of the BRs’ network, using the full extent of existing conserved areas enabled a more comprehensive evaluation. This approach positions the WNBR to strategically complement the broader conservation landscape, identifying underrepresented regions rather than simply filling BR-specific gaps. The second dimension is conservation value, assessed using a Conservation Value Index derived from the six templates to evaluate each area’s conservation significance.

We computed a rarity-weighted, multi-template overlay index (CVI) at 1-km resolution to summarize conservation opportunity. For each pixel and for each of the six opportunity templates potentially aligned with KMGBF targets (BHs, REs, PDs, TFs, BCs, ILs), we recorded a binary indicator ${R}_i$ denoting whether the pixel falls within the hotspot mask of template *i* (1 = inside; 0 = outside). To reflect template rarity, we assigned a weight ${w}_i$ to each template that is inversely related to the global land-area share covered by that template’s hotspot mask (${p}_i$); all weights were normalized to sum to one across templates. The pixel-level CVI was then obtained by summing the weights of all templates that the pixel ‘hits’. Intuitively, CVI increases when a pixel intersects more templates and/or intersects rarer templates. For visualization and sextile binning in the 6 × 6 quantile matrix, we applied a min–max standardization of CVI across all terrestrial pixels. After calculating the CVI, we applied min–max normalization to each global cell. Finally, we prioritized post-2025 WNBR expansion to Udvardy’s Biogeographical Provinces that combine high conservation value and low existing coverage, operationalized as provinces in the upper-value (Q5–Q6) and lower-coverage (Q1–Q2) sextiles of the 6 × 6 quantile matrix, where Q1–Q6 denote empirical quantile bins (sextiles) from lowest (Q1) to highest (Q6) for each variable. The formulas are given below:


(1) \begin{eqnarray*}
{w}_i = \frac{{\frac{1}{{{p}_i}}}}{{\mathop \sum \nolimits_{j = 1}^n \frac{1}{{{p}_j}}}},\quad \mathop \sum \limits_{i = 1}^n {w}_i = 1,
\end{eqnarray*}

(2) \begin{eqnarray*}
{\mathrm{CVI}} = \mathop \sum \limits_{i = 1}^n {w}_i\,{R}_i,
\end{eqnarray*}

(3) \begin{eqnarray*}
{\mathrm{CV}}{{\mathrm{I}}}_{{\mathrm{std}}} = \frac{{{\mathrm{CVI - min(CVI)}}}}{{{\mathrm{max(CVI) - min(CVI)}}}},
\end{eqnarray*}

where *i* represents the index of opportunity templates (here *n* = 6: BHs, REs, PDs, TFs, BCs, ILs); ${R}_i \in {\mathrm{\{ 0,1\} \ }}$represents pixel membership in template *i* (1 = inside hotspot mask; 0 = outside);${p}_i \in {\mathrm{(0,1]}}$ represents global land-area share covered by template *i*’s hotspot mask;${w}_i\ $represents rarity-based normalized weight; ${\mathrm{CVI}} \in {\mathrm{[0,1]}}$ represents rarity-weighted multi-template overlay index; ${\mathrm{CV}}{{\mathrm{I}}}_{{\mathrm{std}}} \in {\mathrm{[0,1]}}$ represents min–max standardized CVI across all terrestrial pixels.

To assess human pressure across Udvardy’s Biogeographical Provinces, the global HFP dataset [79] was used. HFP scores integrate multiple anthropogenic factors including built environments, population density, nighttime lights, crop lands, roads, railways, and navigable waterways. The mean HFP value for each biogeographical province was calculated by overlaying the HFP layer (year 2020, at 1 km resolution) with the spatial boundaries of Udvardy’s provinces. Zonal statistics were performed and the results were aggregated to visualize the spatial distribution of mean HFP scores across provinces.

## Supplementary Material

nwaf449_Supplemental_File

## Data Availability

Information on the World Network of Biosphere Reserves was obtained from UNESCO’s MAB official website (https://www.unesco.org/en/mab/map) and the biodiversity portal (https://biodiversity.unesco.org/portal/sites). Data on protected areas and other effective area-based conservation measures were derived from the World Database on Protected Areas (https://www.protectedplanet.net/en) and the Chinese National Earth System Science Data Center (https://www.geodata.cn/aboutus.html). The shapefiles of BRs used in this study are available from the corresponding author upon request.
