# Supplementary material for: Potential of the World Network of Biosphere Reserves to advance the Kunming-Montreal Global Biodiversity Framework
Source: Natl Sci Rev. 2025 Oct 21;12(12):nwaf449. doi: 10.1093/nsr/nwaf449 (PMC12707068; doi:10.1093/nsr/nwaf449)
Supplement: nwaf449_Supplemental_File [file nwaf449_supplemental_file.docx]

*Supplementary Information for*

**Potential of the World Network of Biosphere Reserves to advance the Kunming-Montreal Global Biodiversity Framework**

**
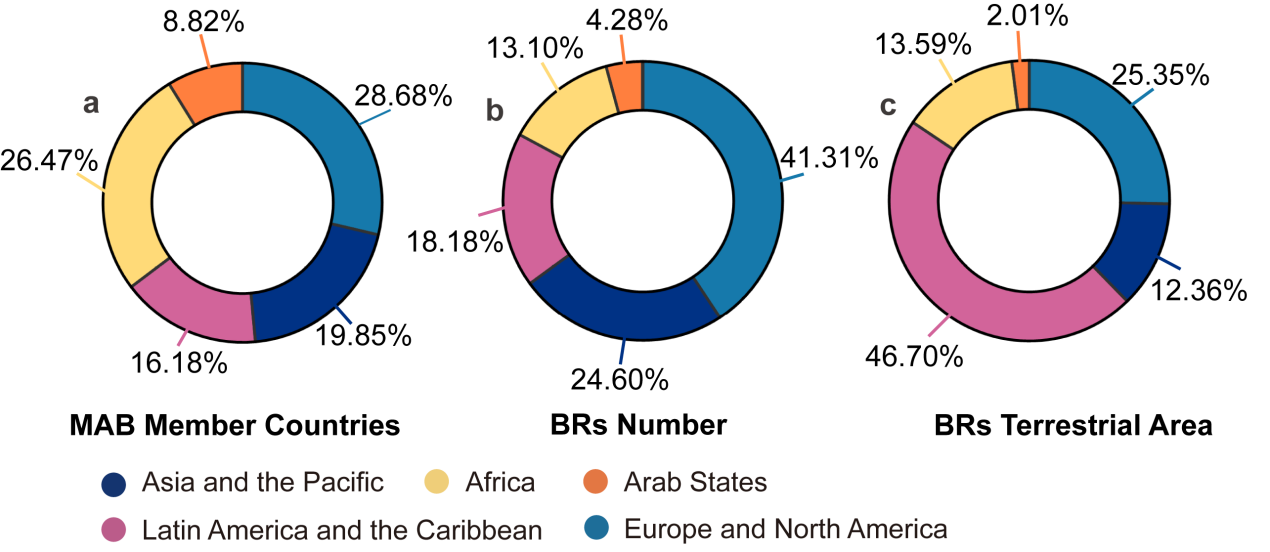
**

**Supplementary Fig. 1.** Distribution of BRs across different regional groups. **(a)** The proportion of MAB member countries within each regional group. **(b)** The percentage of BRs designated in each region relative to the total number of BRs worldwide. **(c)** The percentage of total terrestrial area covered by BRs in each region relative to the global BR terrestrial area.


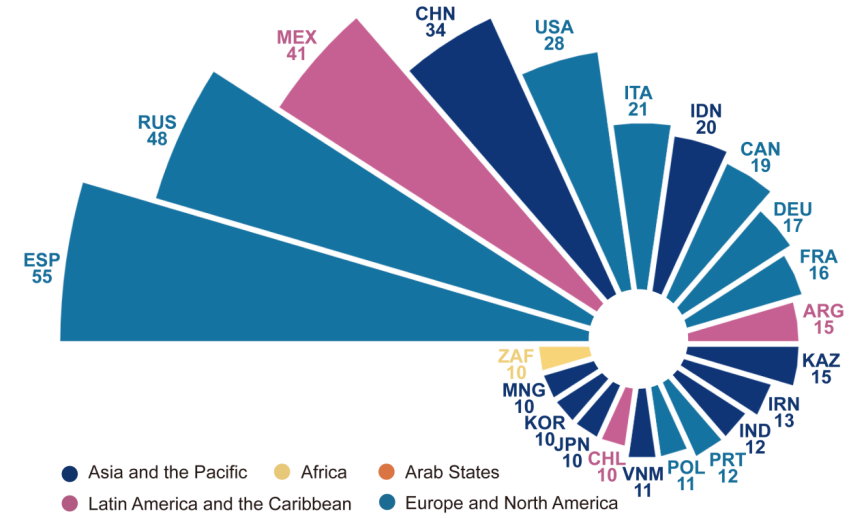


**Supplementary Fig. 2.** Countries ranked by the number of BRs (only countries with 10 or more BRs are shown in the map)

**
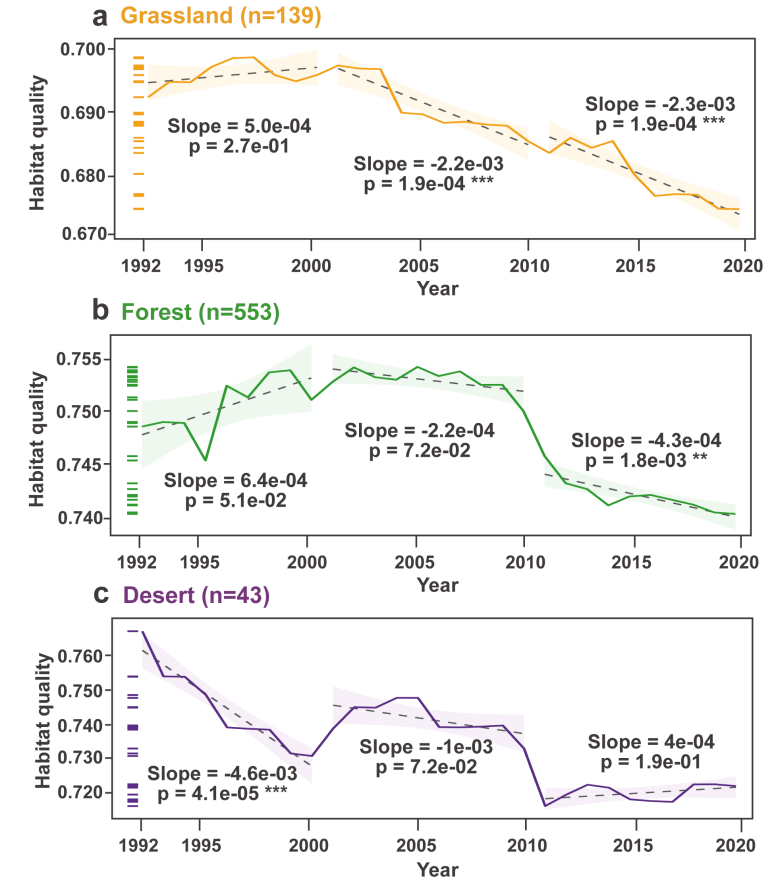
**

**Supplementary Fig. 3.** Temporal trends in average habitat quality among BRs from 1992 to 2020 categorized by ecosystem type: **(a)** Grasslands, **(b)** Forests, and **(c)** Deserts. Slopes and p-values for each period (1992–2000, 2001–2010, and 2011–2020) are indicated in each panel to illustrate temporal trends. The significance levels are denoted as **p* < 0.05; ***p* < 0.01; ****p* < 0.001. Shaded areas represent 95% confidence intervals, and dashed lines indicate the linear regression slopes. Small tick marks on the left side of each panel denote the distribution of annual mean habitat quality values over time.

**
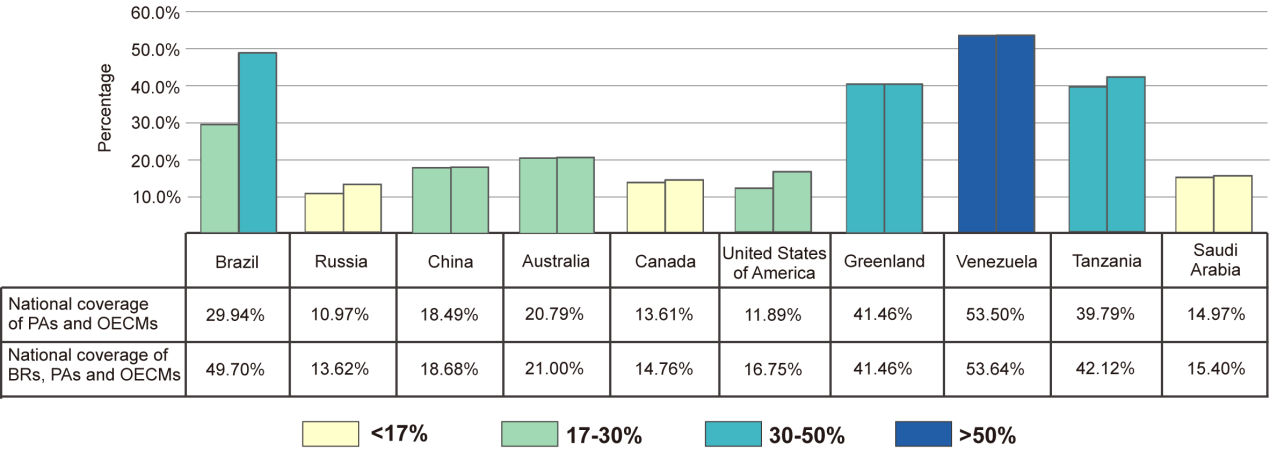
**

**Supplementary Fig. 4.** Bar chart of the 10 countries with the largest PA+OECM area, with percent range conserved shown before and after adding BRs

**
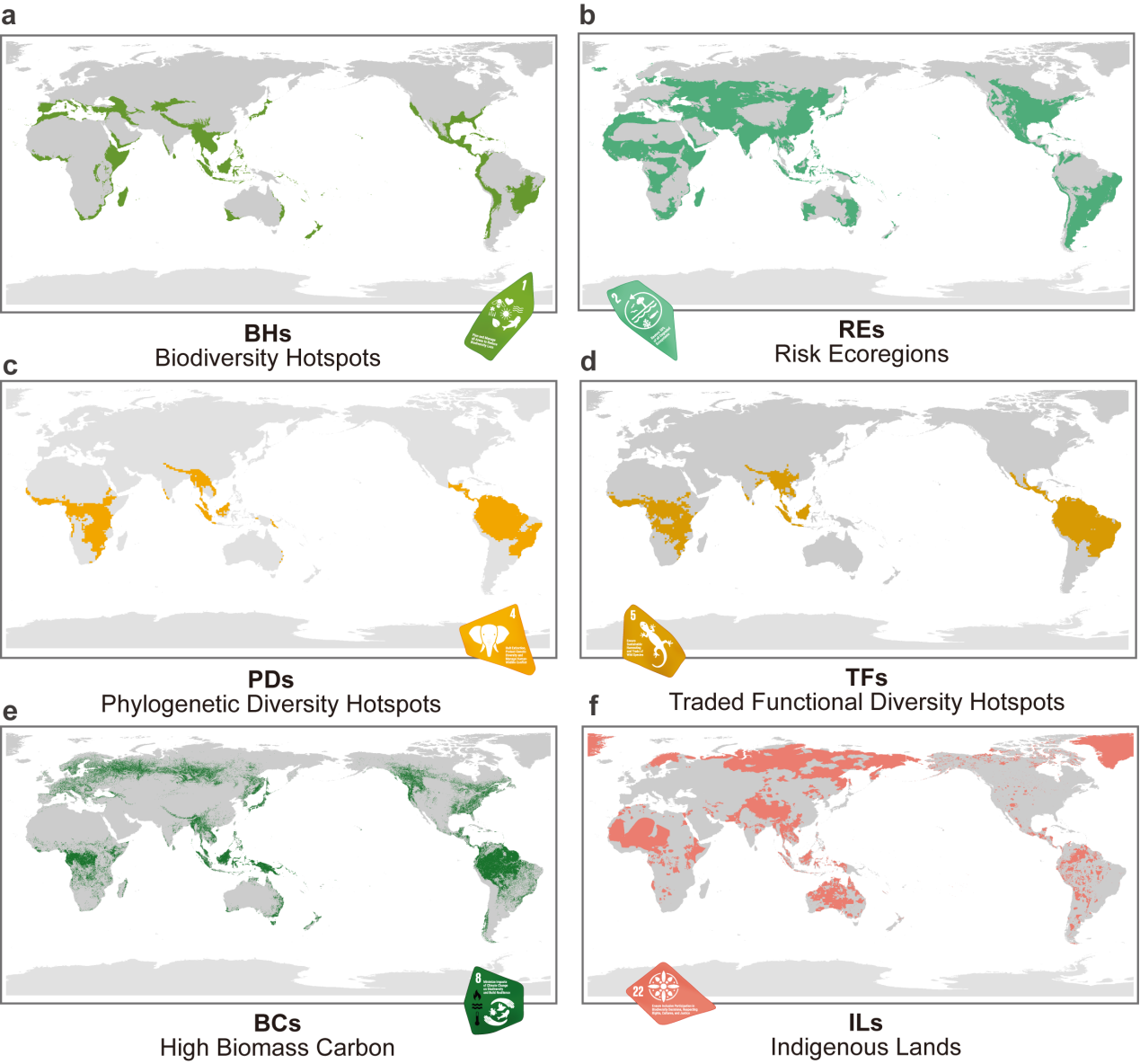
**

**Supplementary Fig. 5. Spatial distribution of six global conservation planning templates used in this research to evaluate the WNBR’ potential in advance the six KMGBF targets.** **(a)** Biodiversity hotspots (BHs)^1^; **(b)** Risk ecoregions (REs)^2^; **(c)** Phylogenetic diversity hotspots (PDs)^3^; **(d)** Traded functional diversity hotspots (TFs)^3^;  **(e)** High Biomass Carbon (BCs)^4^; **(f)** Indigenous Lands (ILs)^5^.

**
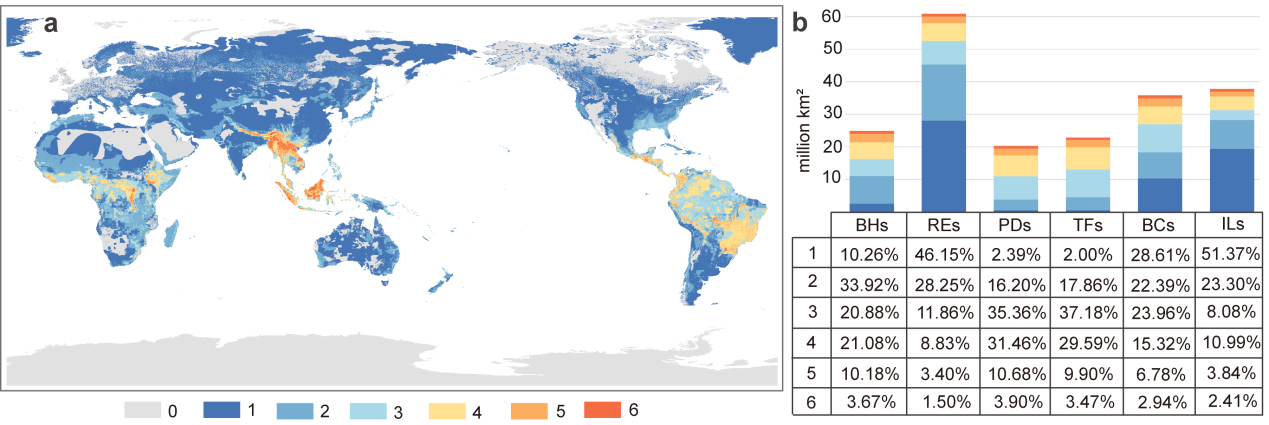
**

**Supplementary Fig. 6.** Spatial meta-analysis results of the six global conservation templates. **(a)** Map showing the frequency of overlap for each 1km × 1km grid, indicating how many of the six templates include the same area (ranging from 0 to 6). **(b)** Proportional composition of each template based on the number of overlapping templates. Bar heights represent the total area covered by each template, subdivided by the number of overlaps with the other five templates.

**
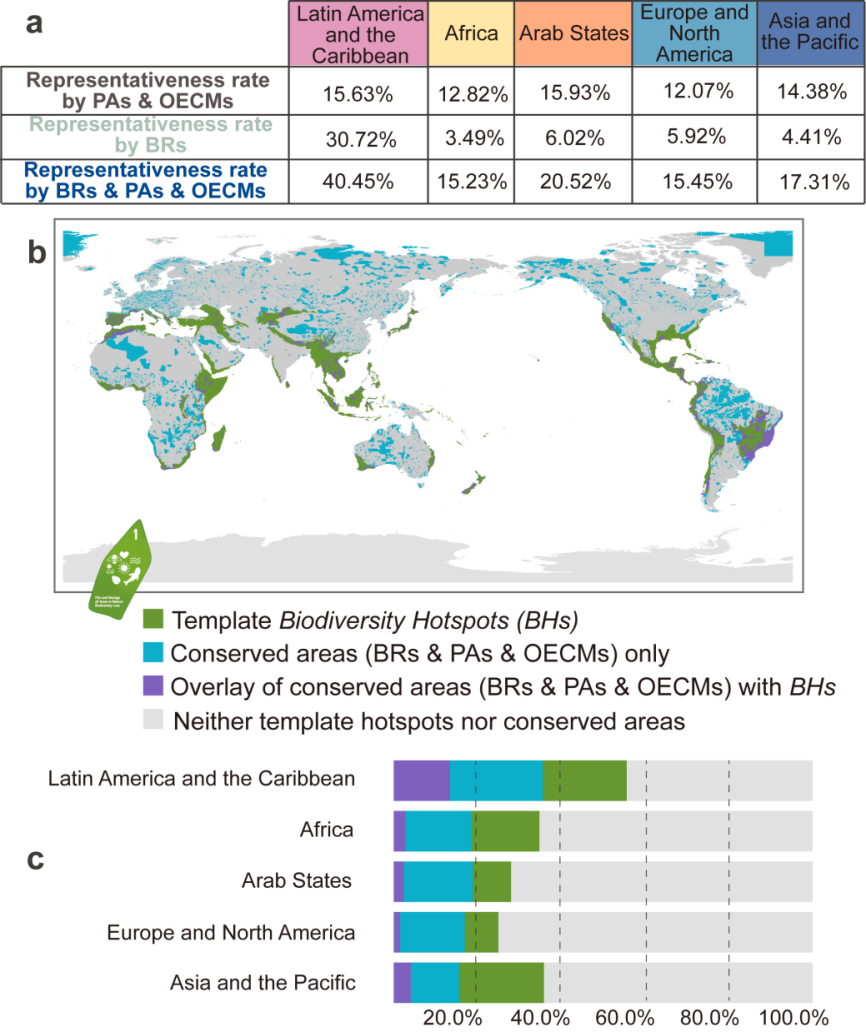
**

**Supplementary Fig. 7.** Representativeness rates of conserved areas for the template Biodiversity Hotspots (BHs) across 5 global regions. **(a)** Representativeness rates for the BHs across 5 global regions respectively by PAs & OECMs, BRs, and BRs & PAs & OECMs. **(b)** Spatial distribution of the BHs hotspots and conserved areas. **(c)** Representativeness rates of conserved areas for the BHs across 5 global regions. BRs: Biosphere Reserves, PAs: Protected Areas, OECMs: Other Effective Area-Based Conservation Measures.


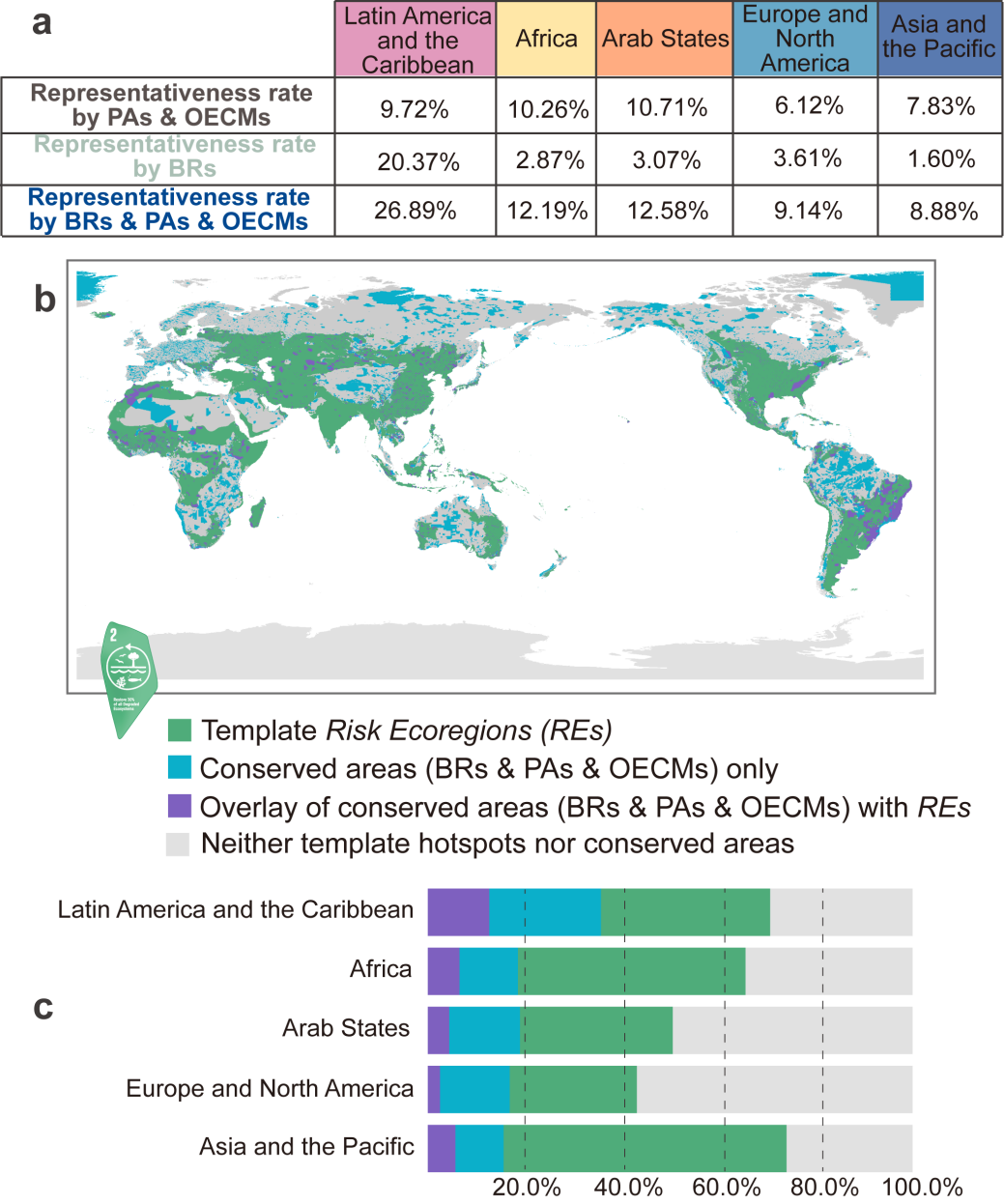


**Supplementary Fig. 8.** Representativeness rates of conserved areas for the template Risk Ecoregions (REs). **(a)** Representativeness rates for the REs across 5 global regions respectively by PAs & OECMs, BRs, and BRs & PAs & OECMs. **(b)** Spatial distribution of the REs hotspots and conserved areas. **(c)** Representativeness rates of conserved areas for the REs across 5 global regions. BRs: Biosphere Reserves, PAs: Protected Areas, OECMs: Other Effective Area-Based Conservation Measures.


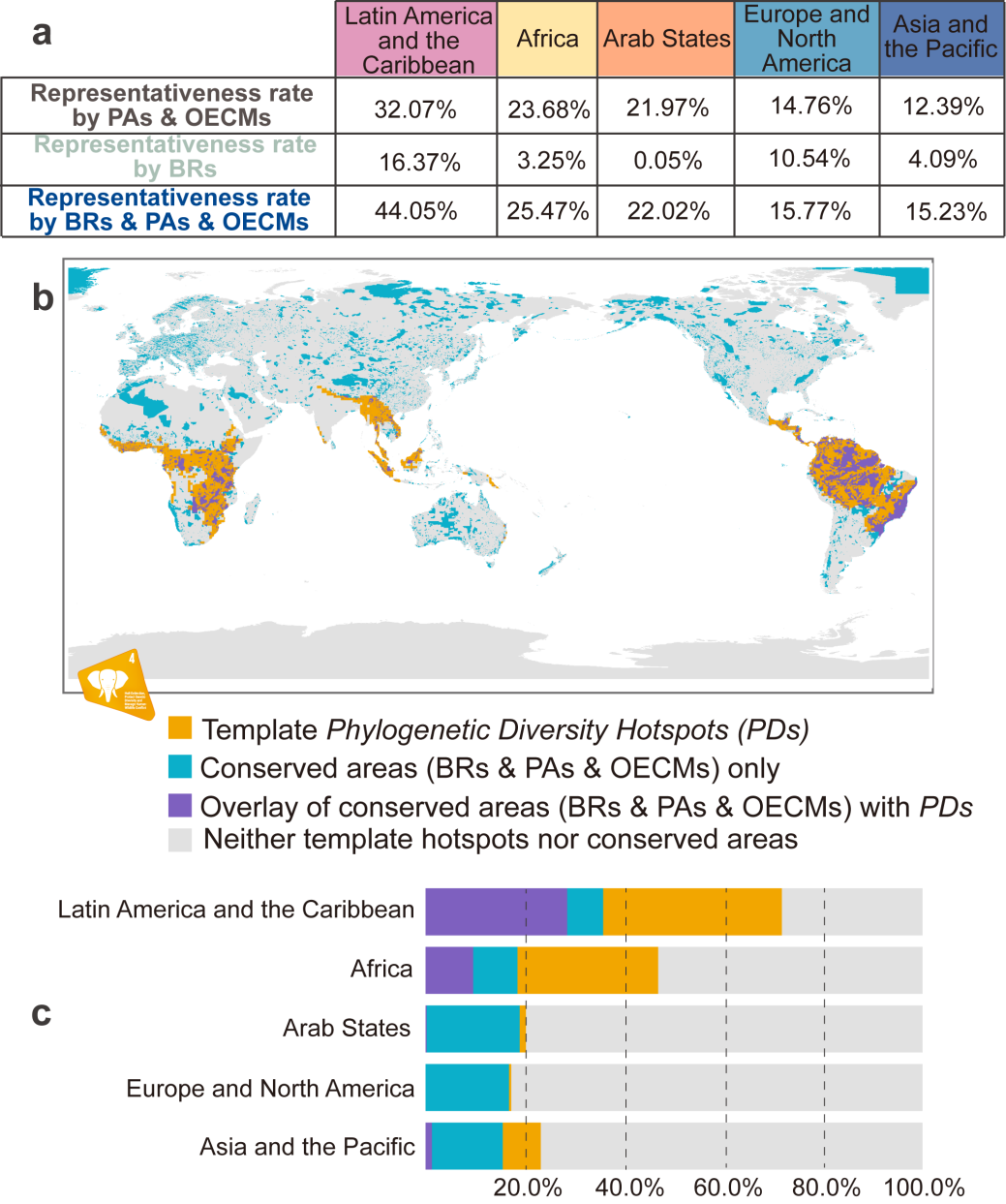


**Supplementary Fig. 9.** Representativeness rates of conserved areas for the template Phylogenetic Diversity Hotspots (PDs). **(a)** Representativeness rates for the PDs across 5 global regions respectively by PAs & OECMs, BRs, and BRs & PAs & OECMs. **(b)** Spatial distribution of the PDs hotspots and conserved areas. **(c)** Representativeness rates of conserved areas for the PDs across 5 global regions. BRs: Biosphere Reserves, PAs: Protected Areas, OECMs: Other Effective Area-Based Conservation Measures.

10
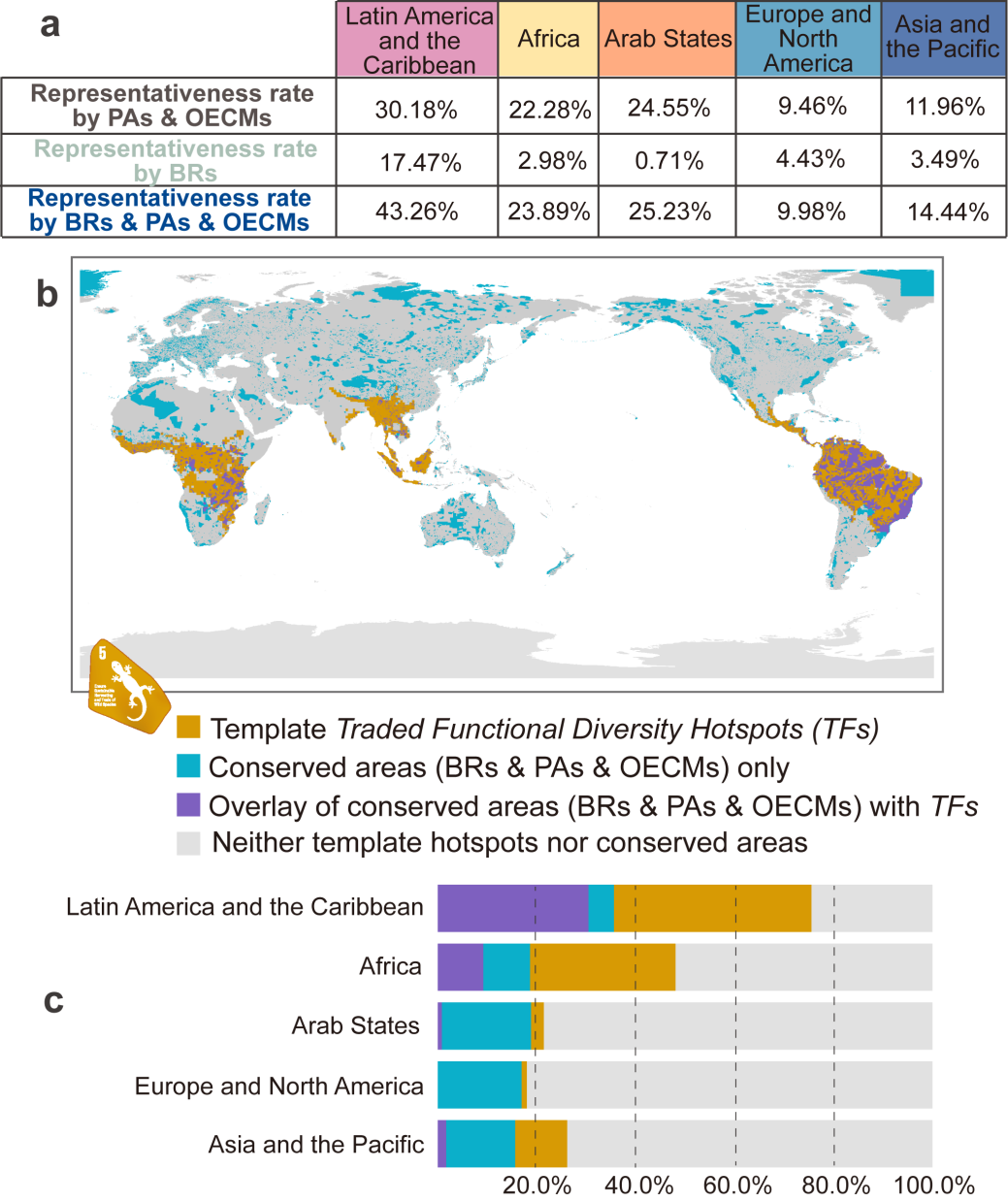


**Supplementary Fig. 10.** Representativeness rates of conserved areas for the template Traded Functional Diversity Hotspots (TFs). **(a)** Representativeness rates for the TFs across 5 global regions respectively by PAs & OECMs, BRs, and BRs & PAs & OECMs. **(b)** Spatial distribution of the TFs hotspots and conserved areas. **(c)** Representativeness rates of conserved areas for the TFs across 5 global regions. BRs: Biosphere Reserves, PAs: Protected Areas, OECMs: Other Effective Area-Based Conservation Measures.


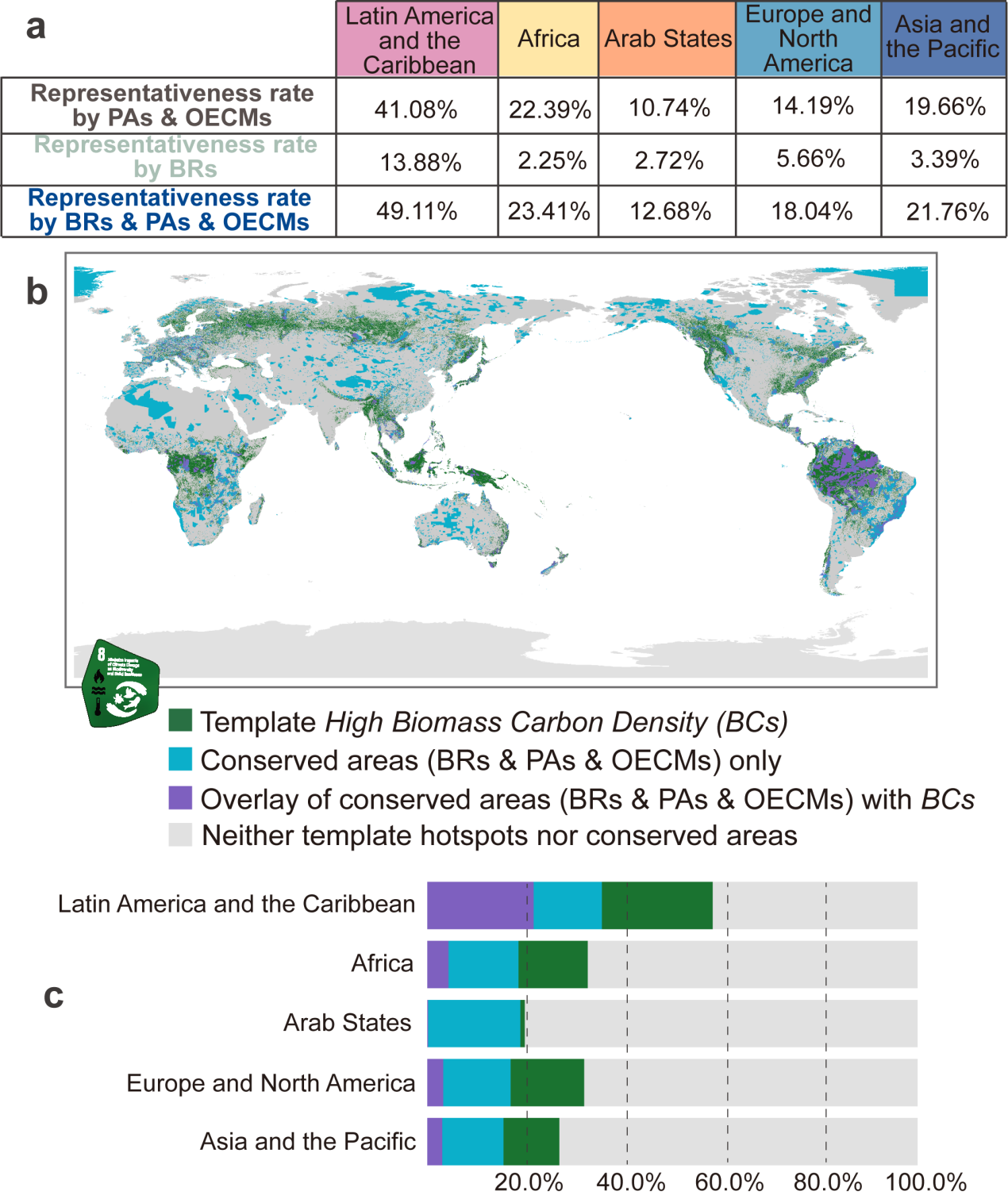


**Supplementary Fig. 11.** Representativeness rates of conserved areas for the template High Biomass Carbon Density (BCs). **(a)** Representativeness rates for the BCs across 5 global regions respectively by PAs & OECMs, BRs, and BRs & PAs & OECMs. **(b)** Spatial distribution of the BCs hotspots and conserved areas. **(c)** Representativeness rates of conserved areas for the BCs across 5 global regions. BRs: Biosphere Reserves, PAs: Protected Areas, OECMs: Other Effective Area-Based Conservation Measures.


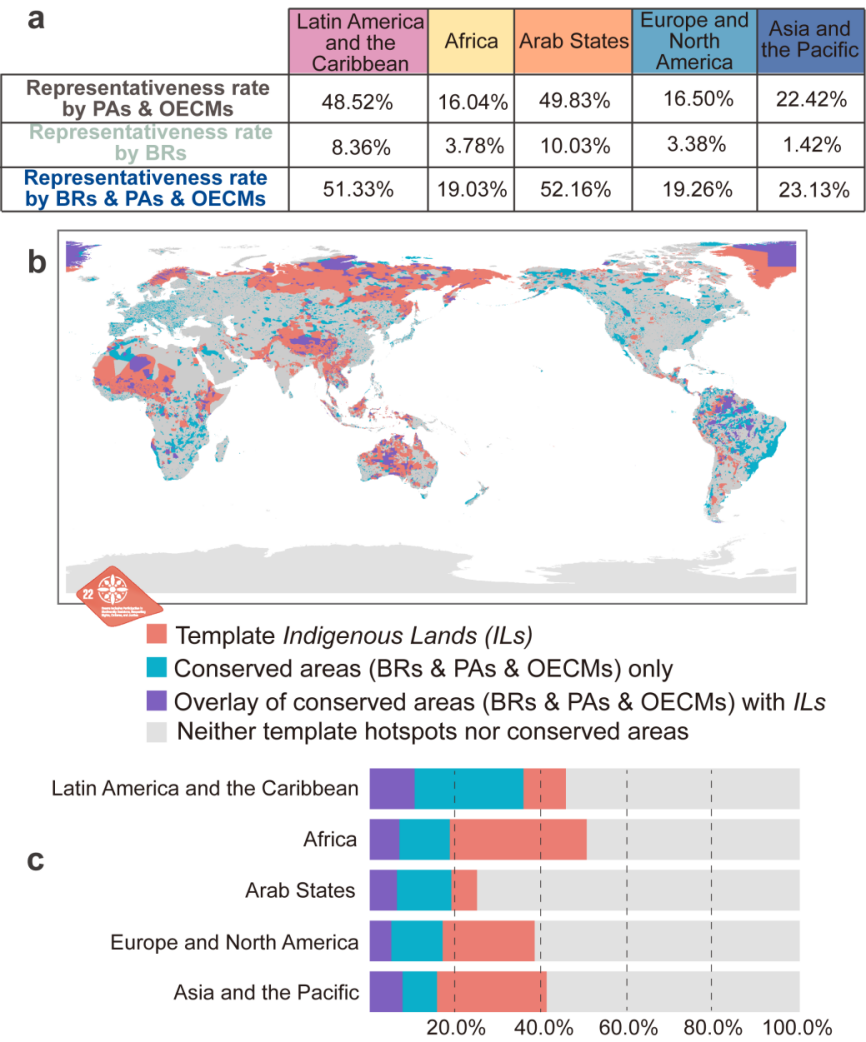


**Supplementary Fig. 12.** Representativeness rates of conserved areas for the template Indigenous Lands (ILs). **(a)** Representativeness rates for the ILs across 5 global regions respectively by PAs & OECMs, BRs, and BRs & PAs & OECMs. **(b)** Spatial distribution of the ILs hotspots and conserved areas. **(c)** Representativeness rates of conserved areas for the ILs across 5 global regions. BRs: Biosphere Reserves, PAs: Protected Areas, OECMs: Other Effective Area-Based Conservation Measures.

**
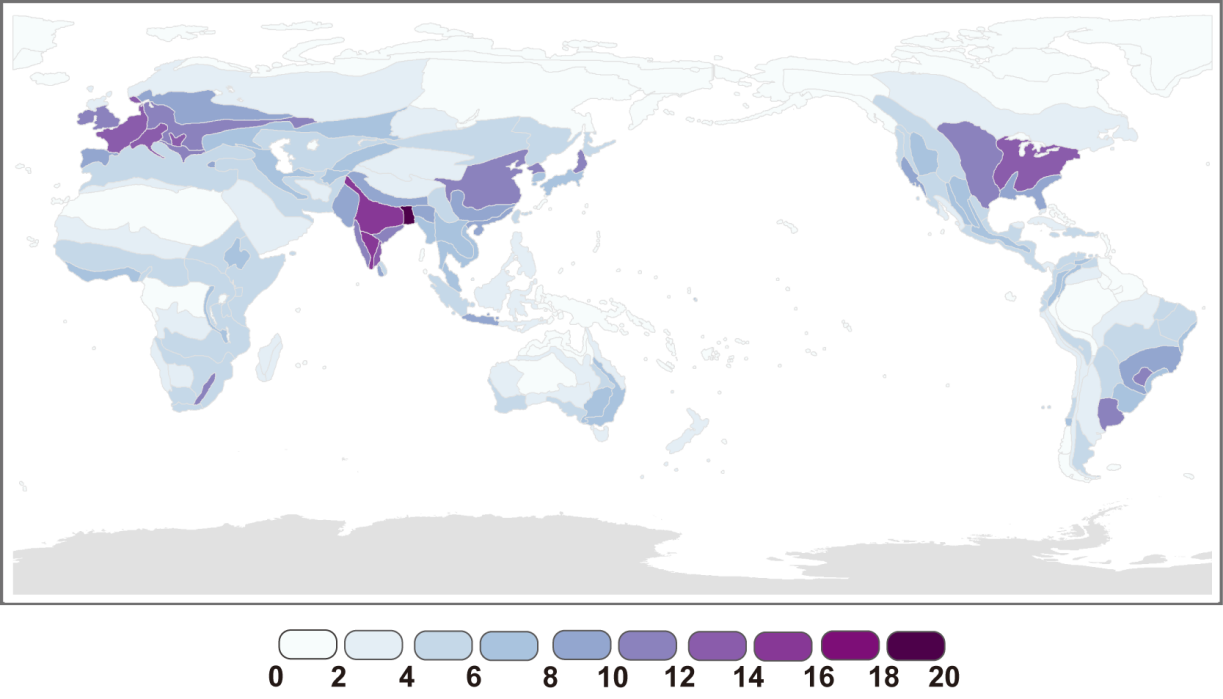
**

**Supplementary Fig. 13.** Mean Human Footprint scores across Udvardy’s Biogeographical Provinces.

**Supplementary Table 1. 17 Udvardy’s Biogeographical Provinces to be prioritized in the future design of the WNBR**

| **No.** | **Udvardy's Biogeographical Provinces** | **Conservation value** | **Current conserved rate (by PAs, OECMs, and BRs)** | **Mean Human Footprint** |
| --- | --- | --- | --- | --- |
| 1 | Andaman and Nicobar Islands | 0.3383 | 0.33% | 0.46 |
| 2 | Austroriparian | 0.3104 | 6.51% | 9.29 |
| 3 | Burma Monsoon Forest | 0.7794 | 4.54% | 8.60 |
| 4 | Burman Rainforest | 0.6888 | 2.52% | 7.26 |
| 5 | Chilean Araucaria Forest | 0.3231 | 0.66% | 7.72 |
| 6 | Congo Woodland | 0.4098 | 9.06% | 3.82 |
| 7 | East Melanesian | 0.2993 | 3.04% | 0.64 |
| 8 | Ethiopian Highlands | 0.3374 | 8.14% | 6.60 |
| 9 | Guerreran | 0.4746 | 5.74% | 5.21 |
| 10 | Java | 0.5572 | 10.58% | 9.56 |
| 11 | Lake Tanganyika | 0.4995 | 3.79% | 0.84 |
| 12 | Lake Ukerewe | 0.3269 | 1.82% | 1.70 |
| 13 | Lesser Sunda Islands | 0.3360 | 12.51% | 2.09 |
| 14 | Malabar Rainforest | 0.3270 | 5.96% | 0.19 |
| 15 | Micronesian | 0.3011 | 4.21% | 11.91 |
| 16 | Southeastern Polynesian | 0.3052 | 4.07% | 0.14 |
| 17 | Yucatecan | 0.3402 | 10.77% | 3.58 |

**Supplementary Table 2. Ecosystem type of BRs and corresponding WWF Biomes**

| **BR’s** **Ecosystem Type** | **WWF Biome** |
| --- | --- |
| Forest | Tropical and Subtropical Moist Broadleaf Forests |
|  | Tropical and Subtropical Dry Broadleaf Forests |
|  | Tropical and Subtropical Coniferous Forests |
|  | Temperate Broadleaf and Mixed Forests |
|  | Temperate Coniferous Forests |
|  | Boreal Forests/Taiga |
|  | Mediterranean Forests, Woodlands, and Scrub |
|  | Mangroves |
| Grassland | Tropical and Subtropical Grasslands, Savannas, and Shrublands |
|  | Temperate Grasslands, Savannas, and Shrublands |
|  | Flooded Grasslands and Savannas |
|  | Montane Grasslands and Shrublands |
| Polar and Tundra | Tundra |
|  | Rock and Ice |
| Desert | Deserts and Xeric Shrublands |
| Ocean | Ocean |
| Freshwater | Fresh water |

**Supplementary Table 3. Appendix S1. Description of IUCN management category**

| **IUCN management category** | **Definition** | **Description** |
| --- | --- | --- |
| I | Ia: Strict nature reserves  Ib: Wilderness areas | Ia: Strictly protected areas set aside to protect biodiversity  Ib: Large unmodified or slightly modified areas with a management objective of preserving their natural condition |
| II | National parks | Large natural or near-natural areas set aside to protect large-scale ecological processes |
| III | Natural monuments or features | Protected areas set aside to protect specific natural monuments or features, typically small in size with high visit value. |
| IV | Habitat/species management area | Protected areas aiming to protect particular species or habitats, where management reflects this priority and regular, active interventions may be needed to address requirements of particular species or to maintain habitats. |
| V | Protected landscape/ seascape | Protected areas where the interaction of people and nature over time has produced a distinct character with significant ecological, biological, cultural and scenic value. |
| VI | Protected area with sustainable use of natural resources | Protected areas conserve ecosystems and habitats together with associated cultural values and traditional natural resource management systems, where low-level non-industrial natural resource use compatible with nature conservation is seen as one of the main aims. |

**Supplementary Table 4. Characteristic of six global conservation planning templates used in this study**

|  | **Templates** | **KMGBF target** | **Format** | **References** |
| --- | --- | --- | --- | --- |
| 1 | **BHs**  Biodiversity Hotspots | Target 1 | vector | Ref.^1^ |
| 2 | **REs**  Risk Ecoregions | Target 2 | vector | Ref.^2^ |
| 3 | **PDs**  Phylogenetic Diversity Hotspots | Target 4 | tabular | Ref.^3^ |
| 4 | **TFs**  Traded Functional Diversity Hotspots | Target 5 | tabular | Ref.^3^ |
| 5 | **BCs**  High Biomass Carbon Density | Target 8 | raster | Ref.^4^ |
| 6 | **ILs**  Indigenous Lands | Target 22 | vector | Ref.^5^ |

# References

1. Myers N, Mittermeier RA and Mittermeier CG et al. Biodiversity hotspots for conservation priorities. Nature 2000; 403: 853–58.
2. Watson JEM, Jones KR, Fuller RA et al. Persistent disparities between recent rates of habitat conversion and protection and implications for future global conservation targets. Conserv Lett 2016; 9: 413–21.
3. Hughes LJ, Massam MR, Morton O et al. Global hotspots of traded phylogenetic and functional diversity. Nature 2023; 620: 351–57.
4. Spawn SA, Sullivan CC and Lark TJ et al. Harmonized global maps of above and belowground biomass carbon density in the year 2010. Sci Data 2020; 7: 289.
5. Garnett ST, Burgess ND, Fa JE et al. A spatial overview of the global importance of Indigenous lands for conservation. Nat Sustain 2018; 1: 369–74.
